# “Blue Light, Camera, Action!”

**DOI:** 10.1021/acscentsci.4c00317

**Published:** 2024-03-13

**Authors:** Paul O’Callaghan, Olof Idevall-Hagren

**Affiliations:** Department of Medical Cell Biology, Uppsala University, Uppsala 75123, Sweden

In this issue of *ACS Central Science*, Jeremy Baskin and his team showcase their latest feat of phospholipase D (PLD) engineering, by adding blue light-dependent activation to their growing toolbox of PLD-based membrane editing techniques.^1^ Previous optogenetic approaches from the group availed of dimerization systems to recruit optoPLD to membranes of interest and more recently applied evolution-informed design strategies to generate more stable superactive PLDs (superPLDs), with enhanced catalytic activity. However, prior to blue light-triggered recruitment, superPLDs fumble around in the shadowy depths of the cytosol where they may randomly edit unintended membrane targets resulting in various nonspecific effects, including artificially high levels of phosphatidic acid (PA) and for some superPLD variants even cytotoxicity.^2^ While from an imaging perspective this technical problem occurs “out of sight”, it clearly has not been “out of mind” for Baskin and his team, and here they outline how they LOVingly silence this unwanted superPLD promiscuity.

**Figure 1:**
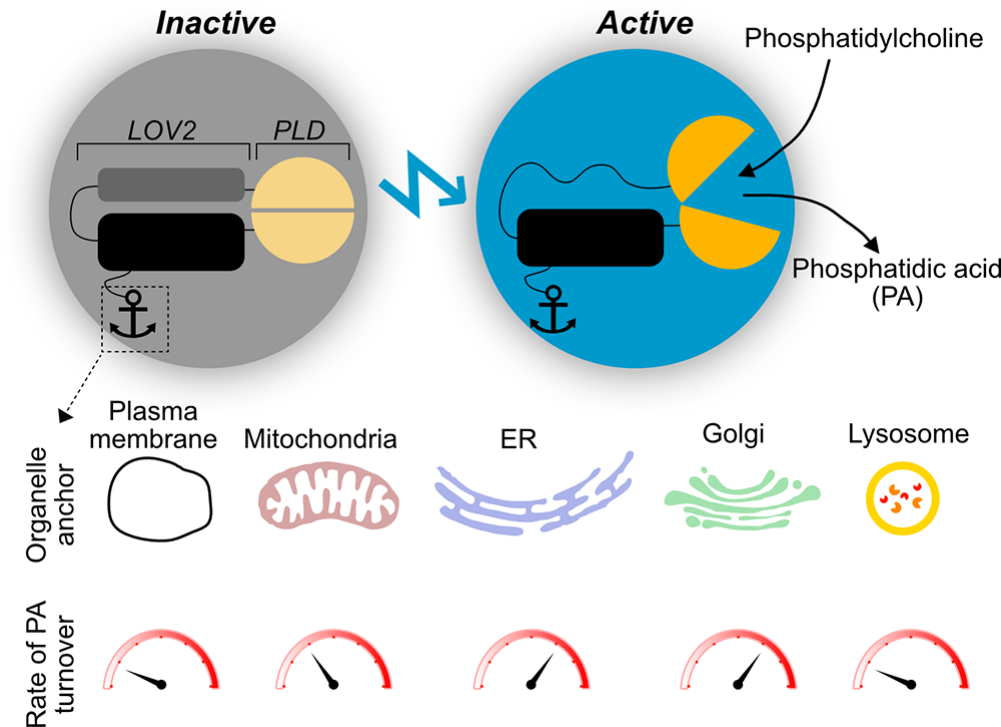
LOVPLD consists of a split PLD that has been fused to the N- and C-terminus of the light sensitive LOV2 domain. This engineered enzyme adopts an inactive conformation in the absence of blue light but rapidly switches to an active conformation upon illumination, which enables rapid hydrolysis of phosphatidylcholine to phosphatidic acid (PA). Fusing different membrane anchors to the LOV2 domain enables specific targeting to organelle membranes, and lipidomic analysis of light-driven PA formation at the different membranes revealed organelle-specific differences in PA turnover rates.

Li et al. sought to address this off-target superPLD activity by editing their editor, through the insertion of the light oxygen voltage (LOV) 2 domain of phototropin (LOV2), whose C-terminal Jα-helix adopts a relaxed conformation in response to blue light. LOV2 domains have previously provided optogenetic regulation of activity-dependent conformations to provide control over the activity of specific signaling pathways, such as Rho-GTPase signaling in studies of cytoskeleton dynamics.^3^ Here, Li and colleagues needed to strike a happy balance between selecting an insertion site for the LOV2 domain that would sterically obstruct superPLD activity in the dark but restore PLD activity once exposed to blue light and so tested inserting the domain into different flexible loop regions of PLD predicted to tolerate its presence. To further constrain the lipid-editing potential of this LOVPLD specifically to the plasma membrane (PM), they added a truncated Lyn anchor and for imaging purposes included an mCherry tag. To quantitatively assess the blue-light stimulated activity of the resulting panel of LOVPLD variants, they took advantage of their IMPACT labeling system, which exploits the fact that in the presence of primary alcohols PLD (in addition to hydrolysis) will catalyze transphosphatidylation reactions to produce phosphatidylalcohols. This membrane modification produces a “clickable lipid” to which fluorescent reporters can be bound and quantified.^4^ The best of this LOVPLD bunch was a variant with no background activity, but when blue-light triggered snapped into action, producing a photoswitchable PLD whose activity reached approximately 50% of that of its superPLD parent and could be controlled in space and time.

## Different organelles have different appetites for PA!

The authors next challenged their LOVPLD assay to explore the nature of lipid metabolism at the membranes of specific organelles. They swapped out the PM-targeting tag and generated LOVPLDs that tethered to the mitochondria, endoplasmic reticulum (ER), lysosomes, and Golgi. IMPACT labeling revealed near similar levels of PLD activity for LOVPLD at each organelle. The authors rationalized that this similarity was to be expected as phosphatidylcholine (PC), the major substrate for PA, represents 50% of lipids in the majority of organelle membranes; therefore, comparable levels of PA generation would be predicted at each site. However, lipidomic analysis did not immediately permit such a straightforward interpretation, as while PA levels were enriched in lysosomes (and at the PM), a similar increase was not so apparent in mitochondria, ER, and Golgi. As previous work indicates that the transphosphatidylation activity reported by IMPACT is a good proxy for PC hydrolysis to PA, the authors considered that LOVPLD’s hydrolytic activity should be intact at each organelle and delved deeper into their lipidomic data. They established that following blue-light stimulation, the relatively lower increase in PA on the ER was accompanied by increased levels of PA derivatives and concluded that the rate and manner in which PA is consumed (or cleared) reveals organelle-specific dynamics.

## “PA on the mitochondria really brings out the kinase in me,” says LKB1

To really put LOVPLD through its paces and showcase the advantages of this membrane editor, Li et al. set out to assess if generating PA on different organelle membranes impacts liver kinase B1 (LKB1) recruitment and whether this in turn alters the capacity of the kinase to activate the metabolic master-regulator AMPK. Imaging of GFP-tagged LKB1 revealed a blue-light stimulated shift of signal from the nucleus to regions enriched for each of the organelle-targeted LOVPLD constructs, indicating that kinase recruitment was dependent on LOVPLD-mediated PA generation. The authors note that a similar GFP-LKB1 enrichment was not observed for PM-targeted LOVPLD, identifying a membrane specific component to the PA-dependent recruitment of LKB1. To isolate the effects on AMPK, they coexpressed either a cytosolic or ER-targeted variant of an AMPK activity reporter with their PM- and organelle-targeted LOVPLDs. Blue light stimulation significantly increased both cytosolic and ER-bound AMPK activity in cells expressing mitochondrial-targeted LOVPLD, and while activated ER-targeted LOVPLD increased cytosolic AMPK activity, it surprisingly did not increase the activity of ER-bound AMPK. Disentangling the implications of these results will no doubt serve as the focus of future studies from the lab, but at the very least these experiments highlight the complexity of interorganelle lipid-dependent communication, which will now be amenable to experimental interrogation thanks to new and improved membrane editing tools like LOVPLD.

The current study clearly shows the potential for engineering lipid metabolizing enzymes to control the concentration of specific lipids with high spatial and temporal precision, and this methodology may be broadly developed to probe the function of other, even less-characterized lipids. The LOV domain, used here to control enzyme activity, obtains an open conformation in the presence of blue light (400–500 nm) and rapidly inactivates in the absence of blue light. These two characteristics present some technical challenges; first, to maintain LOVPLD activity for prolonged periods requires continuous or repetitive blue light illumination. While the light-intensity required for the latter is likely low, the total light exposure during prolonged experiments may still present a risk for phototoxicity.^5^ A possible solution to this could be to employ LOV domain variants with much slower inactivation kinetics that might enable prolonged enzyme activity to be induced by a single light pulse. Another approach could be to combine a slow-inactivating LOV domain with a red-light driven dimerization system, such as BphP1-PpsR2,^6^ or to take the spatial limitation of its activity toward single molecule resolution by combining it with the expression of light-generating luciferase enzymes.^7^

A second issue is that the absorption spectrum of the LOV domain overlaps with commonly used fluorophores, such as GFP and YFP, thereby complicating protocols requiring LOVPLD activation and simultaneous imaging of a tagged target of interest. If the LOV domain could be engineered to relax in response to red-shifted light absorption, it would minimize the risk and may also open up for the possibility of in vivo activation experiments, thanks to the deeper penetration of red light. However, this will likely be challenging since the LOV absorption is defined by oxidation of a flavin cofactor and not directly by the peptide backbone. Baskin and his team will undoubtedly apply creative engineering solutions to further increase LOVPLD’s utility in future iterations, but from a biological point of view, this new generation of lipid editor already reveals the unexpected organelle-specific regulation of PA that operates on the time scale of hours and likely involves secondary metabolism of the lipid. PA also takes part in rapid signaling that controls biological processes like endocytosis and exocytosis on a time scale of seconds to minutes. Although not explored in the current study, the tools developed by Li and colleagues are ideally suited to facilitate future studies aimed at deciphering these and other aspects of PA signaling.
